# The effects of AI-collaborative translation workshop on translation competence and learner engagement: an empirical study in a Chinese university translation course

**DOI:** 10.3389/fpsyg.2026.1918404

**Published:** 2026-08-20

**Authors:** Weihe Xue

**Affiliations:** School of Foreign Languages, Fujian Business University, Fuzhou, China

**Keywords:** AI-collaborative translation workshop, Chinese university students, Chinese-English translation, learner engagement, translation competence development

## Abstract

As artificial intelligence (AI) is integrated into translation pedagogy, it brings new possibilities for competence development. However, concerns also emerge regarding learner engagement and maintenance of human agency. This study investigates the effects of an AI-Collaborative Translation Workshop on translation major students’ learner engagement and translation competence with a mixed-methods approach. 69 undergraduate translation-major students participated in a 16-week quasi-experimental design. The experimental group (*n* = 35) attended a structured AI-collaborative workflow, while the control group (*n* = 34) took a conventional translation course concurrently. Through pre- and post-intervention scales and translation competence tests, quantitative data were gathered, while classroom observations and semi-structured interviews generated qualitative data. According to the ANCOVA results, significantly greater improvements in overall translation performance and engagement were observed in the experimental group. Specifically, the intervention yielded moderate-to-large effects on textual faithfulness and linguistic appropriateness, but the effectiveness of cultural transmission showed no pronounced improvement. In terms of learner engagement, the intervention exerted moderate positive effects on overall engagement, with considerable improvements in behavioral and emotional engagement. No significant change was found in cognitive engagement. Qualitative data corroborated these findings. Students in the experimental group demonstrated more proactive behaviors and positive emotions. Overall, despite strengthening behavioral and emotional participation as well as certain aspects of translation competence, AI-human collaboration still had limited ability to cultivate deep cognitive engagement and culturally sensitive translation skills. Therefore, pedagogical designs should position AI as a resource for critical reflection rather than a substitute for translator decision-making, thereby preserving learner agency and promoting the development of higher-order thinking.

## 1 Introduction

Learner engagement, as a key determinant of academic accomplishment and educational quality, comprises cognitive, emotional, and behavioral engagement (Fredricks et al., 2004; Kuh, 2009; Hiver et al., 2021; Carver et al., 2021). Svalberg (2009) put forward the concept of Engagement with the Language (EWL), including cognitive, social, and affective dimension. This construct explains why certain language-related behaviors and attitudes seem more conducive to learning than others. Language educators have been striving to promote learner engagement. However, many instructors, especially in EFL settings, still struggle to promote sustained learner engagement in practice (Liu, 2022). This challenge is particularly evident in Chinese higher education, where opportunities for authentic engagement tend to be limited by exam-oriented culture and teacher-centered pedagogical traditions (Deng and Carless, 2010).

This issue is evident in translation education. Deemed as linguistic competence earlier, translation now demands skills in cultural, technological, and professional dimensions (Madkour, 2018; Kornacki, 2018; Asiri and Metwally, 2020; Han, 2020). To develop such competence requires active, collaborative, and critically reflective learning environments. In China’s traditional translation classrooms, instructors typically compare students’ outputs with a “standard” version, and offer feedback on errors and shortcomings (Ren, 2022). Under such instructional practice, students’ creative exploration and learner autonomy are restricted. It also hampers long-term task engagement, and ultimately hinders the development of translation competence.

The rise of Artificial Intelligence (AI) has brought pivotal changes in teaching practices (Creely and Carabott, 2025), which enables interactive, participatory student-centered environments (Meng et al., 2025). In translation pedagogy especially, AI tools offer students the opportunities for process-based learning, since routine linguistic tasks are automated by the machine. Moreover, the technology can cultivate a sense of enjoyment in foreign language learning, one of the key drivers for alleviating classroom anxiety and boosting motivation, thereby mitigating the affective barriers common in traditional EFL settings (Liu, 2022). However, there remains a vital constraint. AI translation systems often cannot thoroughly understand the cultural context and implicit meanings embedded in language (Boluwatife, 2025). Indeed, the training of AI models is based on statistical correlations between phrases and words. As a result, they can recognize contextual patterns, but they often overlook subtext, irony, humor, sarcasm, idioms, and metaphors. Therefore, if there is no human intervention, machine-generated translations may result in misinterpretations or cultural inappropriateness.

Although literature on AI in education increases, there is still a notable research gap. Current research has extensively explored the influence of AI translation tools on translation quality and output accuracy (e.g., Hendy et al., 2023; Chan and Tang, 2024; Candé and Martinho, 2026). However, their effects on translation competence and learner engagement within collaborative teaching environments is rarely examined in empirical studies. Most of previous studies viewed AI as an independent tool for product assessment (e.g., Alghamdi and Alotaibi, 2025; Duan et al., 2025), but its mediating role during the in-class learning process was not investigated (Zhu, 2026). Additionally, there is deficient exploration on whether AI-collaborative pedagogy can enhance learner engagement and multidimensional translation competence.

To address this gap, the present study investigates the effects of AI-collaborative instructional intervention on learner engagement in Chinese higher education, specifically on students’ behavioral, emotional, and cognitive engagement. In addition, it identifies the benefits and limitations of using AI in translation teaching. More specifically, it examines whether AI can effectively support the development of students’ translation competence.

## 2 Literature review

### 2.1 Learner engagement

School engagement is traditionally defined as a multidimensional construct, including behavioral, emotional, and cognitive dimensions (Fredricks et al., 2004). Behavioral engagement refers to learners’ positive participation and actions within their learning environment, including attendance, rule compliance, time on task, and involvement in academic-related activities (Fredricks et al., 2004). Emotional engagement reflects learners’ interest and enjoyment in learning tasks (Furlong et al., 2003), as well as how they feel about learning contexts (Libbey, 2004). Cognitive engagement refers to students’ psychological investment and effort in learning, understanding, or mastering knowledge and skills (Newmann et al., 1992).

In the era of Generative AI, it is essential to re-examine the conventional concept of learner engagement. Despite an emphasis on observed participation in earlier studies, recent scholarship focuses on agentic engagement, which is defined as learners’ constructive contribution to the learning process (Reeve, 2013). According to Hiver et al. (2021), engagement is a dynamic process relying on context and action, rather than a static trait. From this perspective, emotions play key roles in regulating learners’ cognitive and motivational resources. In accordance with Control-Value Theory (CVT), achievement emotions affect cognitive resources, motivation, and self-regulation, thereby shaping learning and performance (Pekrun, 2006). These psychological elements are key to sustained learner engagement. This theoretical connection is supported by empirical findings. Kirkpatrick et al. (2026) adopted CVT to demonstrate the role of academic emotions in predicting student engagement and motivation, verifying that emotional appraisals are direct drivers of sustained engagement. Therefore, in the contexts of EFL, viewing engagement in isolation from emotional regulation would be inappropriate, especially in the Chinese EFL context, which is characterized by high anxiety and great effort (Yu et al., 2019). In such high-stakes educational setting, psychological resilience protects learners’ engagement from emotional adversity by helping them tackle the negative influence of academic stress and resource depletion (Wu et al., 2024). Meanwhile, this protective function applies to digital learning contexts, with resilience moderating the connection between basic psychological needs and engagement (Gao et al., 2025). However, internal resources such as resilience may not be sufficient, and external scaffolds may be required to strengthen learners’ sustained engagement.

The introduction of AI and other technologies brings new methods of enhancing learner engagement. Increasing numbers of studies reveal that tools such as ChatGPT, spherical video-based virtual reality (SVVR), and AI-powered translation robots can effectively enhance engagement by providing customized feedback, simulating conversational partners, creating a sense of belonging, and reducing communication anxiety (Zare et al., 2025; Shen et al., 2023; Lei et al., 2025). These findings indicate that proper integration of AI, technology and instruction can help reduce the emotional and motivational obstacles that diminish learner engagement.

To a certain extent, AI can improve learning motivation and efficiency. Nevertheless, it may lead to excessive reliance and cognitive superficiality while undermining independent critical thinking and deep processing (Wang and Wang, 2024; Zhou and Hou, 2024). Therefore, balancing the benefits of technology with chances for autonomous learning of students is crucial during instructional design. From a perspective of engagement, the critical issue lies in the specific conditions and mechanisms behind such effects, rather than whether AI strengthens or weakens learner engagement. This view aligns with the dynamic, context-sensitive definition of engagement applied in this study (Hiver et al., 2021).

Despite extensive study of engagement in general language education, there is insufficient empirical exploration on its specific role in translation learning. Translation is a complicated problem-solving activity, in which persistent cognitive effort is required to deal with linguistic ambiguities, cultural differences, and strategic decision-making (PACTE, 2005). If deep learning is not driven by emotional and behavioral investment, such effort is unlikely to be sustainable (Fredricks et al., 2004; Hiver et al., 2021). In this regard, engagement can be viewed as a motivational core that activates and sustains the cognitive processes needed for translation competence development. Up to now, the engagement-enhancing potential of AI and technology has been explored. Nevertheless, the majority of studies analyze AI as a standalone tool. The impact of AI-collaborative learning on learner engagement has not been fully investigated. In view of these gaps, the present study highlights translation learning as a venue for examining learner engagement in AI-rich contexts.

### 2.2 Translation competence

The notion of translation competence (TC) has developed into a multi-component framework from a bilingual skill. In early research, translation competence was regarded as the bilingualism’s natural extension (Harris and Sherwood, 1978). Then, functionalist or culturalist perspectives influenced scholars to recognize its multi-dimensional nature. PACTE (2005) carried out large-scale empirical research and demonstrated that TC is mainly procedural knowledge. Critically, within the PACTE competence model, bilingual sub-competence and extra-linguistic sub-competence (including bicultural, encyclopedic, and thematic knowledge) are clearly distinguished as two separate dimensions. As suggested by this distinction, translators cannot rely on bilingual competence alone to explain and express culturally embedded meanings. Rather, cultural capacity represents an essential dimension of translation competence. Based on this, Yang and Li (2021) proposed a teaching-oriented model that defined TC as a combination of declarative and procedural knowledge in a particular socio-cultural context. As revealed by these theoretical developments, TC is a learned, multi-faceted ability requiring systematic cultivation.

In the digital era, TC frameworks have extended to incorporate technical literacy, ethical judgment, and cooperative management. Under the 2022 EU EMT framework, TC is divided into five dimensions: personal and interpersonal skills, service provision, language and culture, technology, and translation, with 36 specific skills being involved. In comparison to the 2017 version (European Commission, 2017), the 2022 framework acknowledges the impact of large language models on the industry by adding skills like post-editing machine translation (European Commission, 2022). As reaffirmed by the framework, language and trans-cultural skills are still the key competencies. Beyond that, ethical judgment and human critical thinking distinguish professionals from machines. Moreover, translator’s professional identity in AI-collaborative workflow is redefined through such distinct human cognitive capacities.

In spite of these conceptual improvements, there is still a significant gap in practical evaluation. Cultural transmission has been widely recognized as a key element of TC (Neubert, 2000; House, 2014; Olalla-Soler, 2015); however, it continues to occupy a marginal position in practical translation evaluation. In most existing assessment rubrics, linguistic accuracy and fidelity to source text are prioritized, and cultural transmission is taken as a secondary subjective consideration (Si, 2005; Mekheimer, 2026; Yuan, 2026). Due to such biased evaluation standards, human translators’ key skills, such as the ability to uncover nuanced cultural contexts and to express contextually proper meaning across languages, may be devalued. AI translation lacks such capability. Although TC frameworks increasingly emphasize situated decision-making and procedural knowledge, empirical studies continuously employ product-oriented designs to assess translation quality via final outputs (e.g., Salimi and Shahrestani, 2009; Quinci, 2015). Due to this process-product research gap, how learners engage with tools, peers, and tasks to develop TC cannot be explained.

Empirical studies reinforce the aforementioned concerns. It has been verified that some technological tools can improve the translation efficiency and superficial quality. According to Alkodimi et al. (2024), AI assistance greatly improves the quality of literary translation. Nonetheless, translation tools still face challenges to address in-depth cultural adaptation. Specifically, despite AI’s advantages in language transfer and terminological precision, effectively conveying cultural connotations remains a bottleneck for AI. As pointed out by Shen et al. (2025), machine translation has potential to clarify cultural elements, but it still shows weakness in comparison to professional human translators. Additionally, openness and subjectivity increase the difficulty of fully encoding cultural interpretations into machines. Besides, current courses tend to lag behind industrial demands. Jalambo et al. (2023) argued that Master’s programs lack training in post-editing, project management, and technical skills. According to Zhang and Li (2026), Chinese English learners have found algorithmic bias and academic integrity issues in AI-generated translation, yet their ethical awareness is narrow.

These findings demonstrated that the development of TC, particularly its cultural and ethical dimensions, is a dynamic process requiring persistent engagement with complicated translation tasks, technological mediation, and peer cooperation, rather than just a matter of gaining knowledge or grasping tools. Without such situated and collaborative engagement, learners may fall into cognitive superficiality and over-reliance on AI, and could not develop the vital cultural and ethical awareness needed in the AI era. To narrow this gap, the present study examines how AI-collaborative pedagogical design transforms the engagement processes underlying TC development, and enables the researcher to assess whether the AI-collaborative translation workshop boosts linguistic accuracy and students’ capacity of managing cultural meaning in translation.

### 2.3 AI-integrated translation pedagogy

As AI is integrated into translation education, it has transformed the pedagogical paradigm from a conventional teacher-dominated model to a more dynamic technology-mediated one. Current research has verified the technological efficacy of this shift. Empirical studies consistently show that AI-assisted tools remarkably outperform traditional manual approaches in grammatical correctness, fluency, language precision, and terminology accuracy (Kruk and Kałużna, 2025; Quan, 2026). Furthermore, recent findings suggest that Translation Quality Evaluation feedback produced by Large Language Models (LLMs) shows significant overlap with expert assessments (Jiao et al., 2025), and AI translation tools may facilitate the development of Intercultural Communicative Competence (Mao et al., 2026). As validated by current evidence, AI is a performance enhancer on measurable linguistic indicators, yet the research designs supporting these claims are mainly product-oriented. Owing to this product-oriented focus, learners’ cognitive and behavioral competence development is usually overlooked. It provides restricted insights into the impact of AI integration on the development route of TC.

This gap in process-oriented research is of great significance. Researchers have warned that excessive reliance on AI may cause cognitive superficiality while decreasing independent problem-solving competency (Wang and Wang, 2024; Zhou and Hou, 2024). Zhang and Li (2026) observed a pattern of high dependence accompanied with low engagement among Chinese EFL learners, in which students use AI tools extensively, yet show limited reflective awareness. Hence, despite the function of technology in alleviating linguistic barriers and boosting short-term engagement, over-reliance on translation tools may constrain learners’ long-term language competence development. Meanwhile, their critical thinking skills will be negatively affected (Murtisari et al., 2023; Li et al., 2023; Yudianto et al., 2025).

Despite improvement of product quality, AI brings great risks to learner agency and cognitive depth without proper mediation. Thus, the key challenge lies in whether instructional designs can convert AI from an external performance-enhancing tool into an internal learning mediator, rather than whether AI can output better translations. In prior studies, AI is regarded as an external variable that influences output quality. By contrast, this study holds that a structural workshop design plays a crucial role in transforming AI from a prop into a cognitive partner.

To address the gaps identified in the preceding review, this study conceptualizes the “AI-Collaborative Translation Workshop” as a pedagogical intervention that reshapes learners’ engagement patterns and subsequently facilitates the development of TC. Within this model, AI-collaborative interaction provides students with cognitive scaffolding and affective support, encouraging them to actively evaluate, negotiate, and improve AI-generated translations. These engagement processes are expected to contribute to the development of higher-order translation abilities, including cultural interpretation, strategic decision-making, and critical evaluation of translation outputs. Therefore, learner engagement is conceptualized as a mediating mechanism linking AI-collaborative pedagogy and TC development.

While learner engagement is theoretically linked to deep learning outcomes, its empirical relationship with TC has not been examined. Meanwhile, although TC frameworks now include cultural and procedural dimensions, assessment criteria still prioritize product quality over the developmental processes through which competence is acquired. Additionally, existing research on AI-collaborative instruction predominantly treats AI as an output-enhancement device rather than a pedagogical mediator that may fundamentally reshape engagement itself. This study, therefore, investigates the effects of an AI-Collaborative Translation Workshop on learner engagement and TC, paying particular attention to the effectiveness of cultural transmission. The research questions guiding the study are as follows:

*RQ 1*: To what extent does the AI-mediated workshop environment affect students’ translation competence?

*RQ 2*: How does the AI-Collaborative Translation Workshop influence Chinese EFL learners’ cognitive, emotional, and behavioral engagement?

## 3 Methodology

### 3.1 Participants

The participants involved in this study were 69 third-year translation majors at a university in southeastern coastal China, comprising 15 male students (21.74%) and 54 female students (78.26%). All were taking the same Chinese-English translation course, and had completed the foundational translation courses required by the curriculum. Prior to the intervention, all participants followed an identical syllabus to ensure comparable academic backgrounds. They were assigned to either the experimental group (*n* = 35) or the control group (*n* = 34) based on their existing class rosters via a non-random assignment. Participants demonstrated comparable English proficiency levels based on their National College Entrance Examination (Gaokao) scores (M_experimental_ = 108.18, SD = 11.93; M_control_ = 107.31, SD = 15.12), with an independent samples t-test revealing no significant difference between the groups (*t* = 0.262, *p* = 0.794). No participants withdrew from the study throughout the 16-week intervention.

### 3.2 Instruments

Four instruments were used in this study: (1) an engagement questionnaire, (2) a translation competence test, (3) classroom observation, and (4) semi-structured interviews. These instruments were triangulated to provide a comprehensive understanding of the research variables, with qualitative data serving to interpret the quantitative findings.

*Engagement questionnaire*. The questionnaire, which was adapted from Reeve’s (2013) agentic engagement scale, measured participants’ engagement in the Chinese-English translation course from four aspects: behavioral, emotional, cognitive, and agentic engagement. It included 17 items on a 5-point Likert scale, which ranged from 1 (Strongly Disagree) to 5 (Strongly Agree). The agentic dimension was excluded from the main analysis, as the intervention mainly targeted emotional experience and explicit engagement. Previous studies have validated the questionnaire, showing satisfactory reliability and validity in similar instructional contexts (Hiver et al., 2021). To further ensure content validity, two experts in educational psychology and translation pedagogy were invited to review the adapted questionnaire by affirming its relevance to certain instructional setting. In this study, Cronbach’s alpha coefficients for the dimensions ranged from *α* = 0.725 (emotional engagement) to *α* = 0.850 (behavioral engagement) at pre-test, and from *α* = 0.760 (agentic engagement) to *α* = 0.916 (cognitive engagement) at post-test, all exceeding the acceptable threshold of 0.70 (Nunnally and Bernstein, 1994), thus indicating a high level of reliability. The pre- and post-test questionnaires were administered before and after the 16-week intervention. To mitigate potential response bias, participants were assured that their responses would be confidential, and that their course grades would not be influenced by their answers.

*Classroom observation*. Observation protocols were developed based on Fredricks et al. (2004) engagement framework and adapted to translation teaching. To establish content validity, the protocols were evaluated by two translation teaching experts uninvolved in the instruction based on the research goals. Then the protocols were revised according to experts’ feedback. To enhance intra-rater reliability and reduce observer drift, the researcher underwent a two-week pre-observation training using video recordings of previous translation classes, which included coding practice and debriefing sessions with a senior researcher. During the 16-week intervention, each group was observed formally once every 2 weeks, with each observation spanning a complete 90-min lesson. To mitigate observer bias and the Hawthorne effect, the researcher maintained a low-profile, non-participatory role, with a focus on pre-defined indicators. During each observation, the researcher took field notes to record representative dialogues, critical incidents, and contextual elements associated with student engagement or disengagement in behavioral, emotional, and cognitive dimensions.

*Interviews*. Following the intervention, a semi-structured interview was conducted with eight students (four came from experimental group and four came from control group, with half identified as highly engaged and half as less engaged according to their observed behaviors and questionnaire scores). To reflect varying degrees of learner engagement, the interview participants were selected through purposive sampling. According to engagement framework of Fredricks et al. (2004), the interview protocol was designed, which covered three key dimensions. Before the formal interviews, question clarity was improved through a pilot study. All interviews were carried out in Chinese, audio-recorded, and transcribed word for word.

*Translation competence test*. Two translation tests were created and reviewed by experts to ensure parallel difficulty levels, thereby minimizing testing effects. Students had 20 min to translate a 150-character Chinese text into English. The test items were sourced from the Test for English Majors (Band 8), China’s highest-level English proficiency exam for senior English majors. The TEM-8 translation tasks consist primarily of essays on humanities topics, which are suitable to measure students’ capacity of translating culture-loaded texts. In terms of the TEM-8 scoring standard, there is an emphasis on linguistic appropriateness and textual faithfulness. As House (2014) argued, translation quality assessment should move beyond mere linguistic comparison and address the cultural gaps between the source and target texts. To better assess participants’ competence in translating cultural content, this study added a third criterion, effectiveness of cultural transmission. Textual faithfulness assesses the completeness and accuracy of the translation; linguistic appropriateness evaluates the translation’s grammatical correctness, fluency, and lexical selection; effectiveness of cultural transmission focuses on whether the translation successfully conveys the cultural connotations and pragmatic functions embedded in the source text to the target readers. For instance, translating “压岁钱” as “lucky money” would receive a relatively high score on textual faithfulness and linguistic appropriateness, as the English phrase roughly conveys the idea of money given for good fortune. However, it would score relatively low on effectiveness of cultural transmission, because lucky money fails to convey the specific cultural practice of elders giving money to children during the Spring Festival to ward off evil spirits. The final weighting was distributed as follows: effectiveness of cultural transmission (30%), linguistic appropriateness (30%), and textual faithfulness (40%) (see Appendix B). In the beginning, two raters discussed the scoring standards and conducted a trial scoring on five randomly chosen papers. On this basis, a unified scoring criterion was formed. Furthermore, they independently scored all test papers. Inter-rater reliability was acceptable, with a Pearson correlation coefficient of *r* (69) = 0.946, *p* < 0.001. The average of the two raters’ scores was taken as the final score for each participant.

### 3.3 Procedure

In this study, a pre-post control group quasi-experimental design was adopted. Prior to the intervention, the researcher collected pre-test data from both groups, which included the translation test and the engagement questionnaire. The intervention lasted for 16 weeks, and two sessions (90 min) were held consecutively each week. In the intervention, the experimental group engaged in an AI-collaborative translation workshop, involving AI-generated drafts, human-machine cooperative revision with annotated justifications, and group-based comparative analysis to complete the translation. As for the control group, they followed a general teacher-dominated instructional model. The whole intervention used field notes for structured observation.

After the intervention, this study immediately collected post-test data in the 16th week, including the student interviews, translation post-test, and engagement questionnaire post-test. In the following semester, the control group will receive identical AI-collaborative translation training to address ethical concerns about unequal access to AI tools.

### 3.4 Intervention design

The purpose of instructional intervention was to compare the effects of the AI-collaborative translation workshop and the traditional teaching model on TC and learner engagement. To ensure experimental rigor, the instructional content for both groups was exactly the same, with the same instructor teaching the course.

#### 3.4.1 AI-collaborative translation workshop

The instructional design for the experimental group was grounded on human-AI cooperation and process-centered learning. AI was integrated into students’ translation process. This workshop model did not use machine translation merely as a tool, but established an interactive learning loop via three key stages.

Before each class, the instructor selected thematic texts according to the syllabus, and employed a designated AI tool (DeepSeek in this study) to generate an initial translation. While resolving basic language transfer problems through AI, this step allowed more class time for higher-order tasks.

In class, by reading the AI-generated draft, students identified wrong translations, omissions, or awkward phrasing. Then, they applied translation theories to revise the text. According to the requirements, students were required to clearly justify each modification using the electronic commenting function, such as preserving cultural connotation by using free translation for a culture-loaded term.

Students collaborated in groups for multidimensional comparisons of their revised versions, the original AI draft, and peers’ translations. Furthermore, comparison and negotiation helped groups reach a consensus on translation decisions. Additionally, each group produced a translation log that documented different views and final solutions. As a consequence, a final draft under collective approval was formed.

#### 3.4.2 Control group instruction

The control group followed a conventional teacher-dominated model. Initially, the instructor explained the translation theories and skills for the session, including amplification and omission. In addition, standard or sample translations were provided. On the one hand, the instructor verbally analyzed tactics and skills for processing difficult sentences. On the other hand, students took notes while listening.

Students completed the translation tasks independently during class, relying on print dictionaries and prior knowledge without immediate peer or technological support. After class, the teacher graded assignments using an error-correction approach, identifying grammatical errors, improper word choices, or awkward phrasing, and providing corrected reference translations. Common errors were reviewed at the beginning of the following class.

It should be acknowledged that the instructional intervention integrates AI support with collaborative learning activities like group discussion within the experimental condition. Therefore, the research design does not allow for a complete disentanglement of the unique effects of AI tools from those of collaborative pedagogy.

### 3.5 Analysis

#### 3.5.1 Quantitative analysis

A series of Analyses of Covariance (ANCOVAs) were conducted to compare post-test scores between experimental group and control group. For translation competence, separate ANCOVAs were run for the overall test performance and each of the three sub-dimensions including textual faithfulness, linguistic appropriateness, and effectiveness of cultural transmission. In each analysis, the pre-test score was entered as the covariate, the intervention condition as the fixed factor, and the post-test scores as the dependent variable.

Similarly, separate ANCOVAs were conducted for overall engagement and for each engagement dimension measured by the questionnaire. For each analysis, the pre-test score served as the covariate, the corresponding post-test score as the dependent variable, and the intervention condition as the fixed factor.

To ensure the appropriateness of the analyses, the underlying statistical assumptions, including normality, homogeneity of variances, and homogeneity of regression slopes, were checked prior to performing the ANCOVAs. Partial eta squared (*η*^2^) was calculated to estimate the effect size for all analyses. According to Dörnyei (2007), effect sizes for *η*^2^ are categorized into three levels: small = 0.01, moderate = 0.06, and large = 0.14.

#### 3.5.2 Qualitative analysis

To gain an in-depth understanding of the differences in learner engagement before and after the intervention, the classroom observation records and student interview transcripts were transcribed and coded. The coding scheme employed the behavioral, emotional, and cognitive dimensions as the primary categories (Level 1). The secondary categories (Level 2) were aligned with the engagement questionnaire items.

Two coders (the first author and an associate professor specializing in translation) participated in the coding task (Gass and Mackey, 2017; Miles and Huberman, 1994). Both coders reviewed the observation forms and interview transcripts, focusing on events and statements relevant to engagement. They coded the data independently based on the coding scheme. A random 25% subset of the data was selected for preliminary coding to establish consensus; discrepancies were resolved through discussion, and the scheme was refined accordingly. The refined scheme was then applied to the remaining 75% of the transcripts. The inter-coder reliability between the two coders exceeded 85%.

## 4 Findings

### 4.1 Effects on translation competence

In the pre-test, the control group scored slightly higher than the experimental group across all dimensions, with the exception of the effectiveness of cultural transmission. After the intervention, both groups improved, with the experimental group showing greater gains (control post-test M = 8.31, SD = 2.94; experimental post-test M = 8.96, SD = 3.05).

Levene’s test confirmed the homogeneity of variance assumption for overall performance (*p* = 0.156), textual faithfulness (*p* = 0.227), linguistic appropriateness (*p* = 0.428), and effectiveness of cultural transmission (*p* = 0.728). A series of one-way ANCOVAs were conducted on post-test scores with pre-test scores as the covariate.

*Overall translation performance*. A significant group difference was observed at the post-test stage, with *F* = 9.34, *p* = 0.003, partial *η*^2^ = 0.124. According to Dörnyei (2007) effect size guidelines, the partial eta-squared value of 0.124 represents a moderate-to-large effect.

*Textual faithfulness*. A significant group effect was also found, with *F* = 9.252, *p* = 0.003, partial *η*^2^ = 0.123, representing a moderate to large effect. The experimental group outperformed the control group after controlling for pretest differences.

*Linguistic appropriateness*. The ANCOVA yielded a significant group effect, with *F* = 4.559, *p* = 0.036, partial *η*^2^ = 0.065, which constitutes a moderate effect size.

*Effectiveness of cultural transmission*. No statistically significant group difference was observed for this dimension, with *F* = 1.472, *p* = 0.229, partial *η*^2^ = 0.022, indicating a small effect.

### 4.2 Effects on learner engagement

In the pre-test, the overall engagement levels were roughly similar between the control and experimental groups (control pre-test M = 2.44, SD = 0.47; experimental pre-test M = 2.82, SD = 0.29). After the intervention, there was an improvement in both groups, with the experimental group showing a larger increase (control post-test M = 4.01, SD = 0.32; experimental post-test M = 4.32, SD = 0.33).

Levene’s test confirmed that the assumption of equal variances was upheld (*p* = 0.478). According to ANCOVA results, a significant difference was observed between groups at post-test, with *F* = 6.525, *p* = 0.013, partial *η*^2^ = 0.093, indicating a moderate effect (Dörnyei, 2007). To further comprehend the impact of the AI-collaborative translation workshop on different dimensions of learner engagement, this research integrated qualitative data from classroom observations and interviews with the statistical results.

#### 4.2.1 Behavioral engagement

According to the ANCOVA tests, the intervention exerted a large effect on the experimental group’s behavioral engagement, with *F* = 20.627, *p* = 0.000, partial *η*^2^ = 0.244.

Classroom observations and interview data corroborated this quantitative finding. As revealed by qualitative evidence, the AI-collaborative translation workshop created more chances for active engagement, cooperation, and interaction, thereby promoting students’ behavioral engagement. Classroom observation showed that students in the experimental group demonstrated more proactive behaviors. According to observations notes, students were “sitting in groups, listening carefully to the teacher’s introduction of the AI tools’ operational procedure,” with “many students raising their hands and asking questions.” During the translation tasks, students generated translations through active “use of AI, compared different arrangements of verb,” and “actively shared optimization strategies and methods”.

This pattern was further affirmed by interview data. For instance, Wei (highly engaged, experimental group) said:

We conduct many group discussions, because group cooperation is required to complete our classroom tasks. By discussing with my peers, I could get different new perspectives, which fascinates me a lot (Wei, highly engaged, experimental group, interview).

Similarly, Meng (highly engaged, experimental group) stated:

I always take part in the classroom tasks. I enjoy comparing different AI-generated translations and would like to exchange perspectives with my group members (Meng, high-engagement, experimental group, interview).

In comparison, the control group had less interactive classroom dynamic. Accordin to the observation records, “minimal active interaction” was often mentioned, in which students’ behaviors were primarily limited to “quiet note-taking” and “independent exercise completion.” Highly engaged students in control group maintained traditional behavioral habits such as “carefully taking notes and raising hands to ask questions.” However, their engagement was mainly teacher-directed. Less engaged students in the control group displayed even more passive behaviors. Qing stated she “rarely communicates with classmates and sits alone during group activities,” while Ke admitted, “I never participate in group discussions or communicate with my classmates,” and noted, “I cannot concentrate most of the time; I am often late and zone out in class”.

However, as demonstrated by the qualitative findings, not all students from the experimental group experienced the same positive effect of the intervention. Less engaged students in the experimental group admitted that they passively relied on AI in the learning process. Zhang (less engaged, experimental group) said that she “used AI to translate everything directly without trying independently.” Xin (less engaged, experimental group) stated, “I usually just sit and listen to discussions, occasionally showing agreement. I cannot really come up with any suggestions.” As implied by these findings, although students’ behavioral engagement was commonly improved by AI-collaborative activities, their willingness and ability to engage with AI critically determined their active involvement.

#### 4.2.2 Emotional engagement

Similar to behavioral engagement, ANCOVA results showed that post-test emotional engagement scores differed significantly between the two groups, with *F* = 6.472, *p* = 0.013, partial *η*^2^ = 0.092, indicating a moderate effect.

This quantitative result was further explained by the qualitative findings. According to classroom observations, students in the experimental group experienced a more positive emotional atmosphere characterized by “curiosity and a relaxed classroom atmosphere.” In the experimental group, students tended to view AI as an efficient instrument. Highly engaged students expressed their enjoyment. Meng said, “compared with previous courses, the translation sessions in this semester were more interesting, with my favorite part being the AI comparison.” Similarly, Wei stated, “comparing AI translations and engaging in group discussions which I enjoyed. It provides a more vibrant classroom atmosphere than in traditional translation classes”.

Notably, highly engaged students from the experimental group showed “competence anxiety.” In spite of their positive emotional engagement with the tasks, some highly engaged students worried about their own competence improvement. Meng stated,

Sometimes, I feel anxious and worry about my excessive reliance on the tool and non-improvement of my real translation skills (Meng, highly engaged, experimental group, interview).

Similarly, Wei mentioned,

Sometimes, I worry about my insufficient thinking (Wei, highly engaged, experimental group, interview).

These findings suggest that, the intervention enhanced students’ emotional engagement. At the same time, it also evoked students’ reflection on the potential risks of excessive reliance on AI.

In comparison, the control group showed a “flat” emotional atmosphere. However, for highly engaged students in the control group, their sense of “inner achievement” was strong. They felt “fulfilled” from skill improvement, and they did not express the same anxieties about competence development.

#### 4.2.3 Cognitive engagement

Despite the positive effects on behavioral and emotional engagement, ANCOVA results showed no significant improvement in cognitive engagement, with *F* = 0.028, *p* = 0.868, partial *η*^2^ = 0.000.

The qualitative findings helped explain this result. Although students in the experimental group demonstrated some strategic awareness, their cognitive engagement often remained at a relatively superficial level. For instance, when translating paratactic sentences, one student from the experimental group mentioned: “AI could not translate the original text’s implicit conditional relationship. Adding the conjunction ‘if’ would make the translation more accurate.” However, students did not consistently engage in deeper analysis of linguistic choices or critically evaluate AI-generated translations.

Highly engaged experimental students often used AI as a reference source, but rarely explored the reasons for the differences between their own translations and the AI’s suggestions. Meng said,

When translating serial verb constructions, I followed the teacher’s guidance to differentiate the primary and secondary actions and then combined my draft with the AI’s version to modify sentence structure (Meng, highly engaged, experimental group, interview).

She also mentioned,

I seldom explore reasons behind divergent translation outputs or look for comparative materials (Meng, highly engaged, experimental group, interview).

Less engaged students reflected such tendency more evidently. Zhang mentioned, “I do not think the translation methods the teacher taught me were very helpful. I rely on AI throughout the whole translation process.” Xin also said, “I know too few translation strategies and methods, so it’s hard for me to apply them consciously. Indeed, I just grasp approaches for splitting long sentences”.

In comparison, the control group students, particularly highly engaged students, showed more holistic and autonomous cognitive strategies. Yu (highly engaged, control group) noted, “To tackle long and complex sentences, I first analyze sentence structures and arrange semantic meanings according to the learned methods, and then continue to revise.” Xue (highly engaged, control group) said, “After the first draft, I polish repeatedly until the translation becomes fluent and natural”.

In short, as suggested by the quantitative and qualitative findings, AI-collaborative translation learning failed to automatically boost deeper cognitive engagement, despite strengthening the behavioral and emotional engagement of students. This finding may be due to students’ reliance on AI-generated outputs, instead of active engagement with analytical assessment and reflective learning.

## 5 Discussion

This study investigated the effects of a 16-week AI-collaborative translation workshop on Chinese-English translation performance and learner engagement. As shown by the findings, although overall translation performance and engagement were remarkably reinforced by the intervention, there was not uniform effect across different dimensions. To be specific, the textual faithfulness and linguistic appropriateness of the experimental group students were significantly improved, while cultural transmission competence had no significant improvement. With regard to learner engagement, the intervention exerted a positive impact on behavioral and emotional engagement, yet no remarkable improvement was observed in cognitive engagement. These findings suggest that AI-collaborative translation instruction helps facilitate students’ TC development and engagement, but how learners interact with AI tools, as well as how they evaluate AI-generated outputs determine the achievement of deeper learning outcomes.

### 5.1 Effects on translation competence

The finding shows that students’ overall translation competence has been improved. This finding aligns with the previous findings that AI-assisted translation tools are conducive to effectively strengthening learners’ translation performance (Shu, 2021; Qin and Lu, 2024; Feng and Zhang, 2024; Zhang and Qin, 2025). In this study, students in the experimental group achieved significant improvement in textual faithfulness and linguistic appropriateness. One possible explanation is that AI tools have the capacity to measure semantic similarity and to assess perplexity-based naturalness, which facilitates the remarkable improvements in textual faithfulness and linguistic appropriateness. By providing learners with high-density exposure to target language norms, the tools promote the internalization of linguistic appropriateness via repeated revision and comparison (Qin and Lu, 2024).

A noteworthy finding of the present study is the asymmetric effect across dimensions of TC. While the intervention exerted moderate to large impacts on textual faithfulness and linguistic appropriateness, it did not significantly improve the effectiveness of cultural transmission. This differential effect can be interpreted through the perspective of knowledge typology. Hard translation knowledge such as translation methods, syntactic structures and grammar rules has stable structure and can be easily accessed online. Characterized by subjectivity and ambiguity, soft translation knowledge, including stylistic reproduction, cultural sensitivity, and contextual comprehension, requires sustained interaction and practice (Qin and Ma, 2025). AI can effectively support structurally stable dimensions of translation. Its contribution to culturally sensitive, context-dependent translation tasks may be more limited, however, it may have limited contribution to culturally sensitive, context-dependent translation tasks, because these tasks require human interpretation, contextual understanding, and cultural judgment. Given that culture is inherently open and subjective, machine translation still lags behind human translators in cultural interpretation (Shen et al., 2025). Therefore, AI’s augmentative effect appears stronger when the knowledge is more structural and weaker when the knowledge is more context-dependent.

This result was supported by the qualitative findings. According to the interview responses, students rarely questioned AI-generated translations. As shown by classroom observations, students did not revise substantively, but fully accepted AI outputs, even under insufficient expression of cultural implications. The cultural transmission effectiveness had limited improvement, which may reflect their insufficient engagement in the evaluation and critical analysis processes needed for context-dependent translation tasks. As implied by current studies, in addition to information analysis, critical thinking encompasses the ability to judge proper occasions of AI use (Van Den Berg and Du Plessis, 2023; Walter, 2024). In this study, students in the experimental group did not critically evaluate and correct AI’s cultural defects, but often superficially accepted AI-generated translations. In other words, while AI tools may provide useful linguistic support, their educational value depends largely on whether learners actively analyze, question, and revise the AI-generated outputs.

AI-assisted or technological-supported translation does not automatically trigger the development of higher-level translation capacities. Despite AI’s capacity to reduce linguistic obstacles and to provide effective assistance, learners still need opportunities to make interpretive judgment and strategic decision-making. Therefore, future AI-collaborative translation instruction should not only simply provide access to AI tools, but also integrate structured activities, so that learners can compare, justify, and revise AI-generated translations.

### 5.2 Effects on learner engagement

The intervention exerted a moderate positive impact on learners’ overall engagement. As revealed by the analysis of the sub-dimensions, both behavioral and emotional engagement were remarkably improved, yet cognitive engagement showed no significant change. This finding suggests that, compared with learners’ deeper cognitive involvement, AI-collaborative instruction may more readily influence their visible engagement and emotional experiences.

The improvement in behavioral engagement may be attributed to AI’s ability to transform traditional unidirectional knowledge transmission into multimodal interactive learning, which actively promotes students’ behavioral engagement (Shu, 2021). AI provides simulated practical contexts, AI peers, and resource libraries, reshaping the learning process and enhancing learners’ curiosity and engagement (Zhang and Qin, 2025). Driven by these benefits, students could shift from peripheral engagement to comprehensive engagement. According to classroom observations in this study, students in the experimental group were more willing to engage in discussions, exchange their ideas, and participate in comparison activities. These findings indicate that AI tools may increase opportunities for active participation and interaction to augment the behavioral engagement of students.

Likewise, the intervention exerted a positive impact on emotional engagement. As revealed by qualitative data, students tended to regard AI-collaborative activities as interesting and inspiring ones, especially the AI translation comparison practices. This finding aligns with previous research result that AI tools are likely to decrease anxiety and other negative emotional experiences (Kruk and Kałużna, 2025). CVT holds that external scaffolding can protect or boost students’ learning engagement by regulating their negative emotions (Pekrun, 2006). In this study, the AI-collaborative translation workshop offered students such external scaffolding to help maintain their engagement and control their negative emotions.

Notably, there was no significant improvement in cognitive engagement. Although students in the experimental group became more proactive and emotionally engaged, increased participation did not always suggest more in-depth cognitive processing. One possible explanation is that some students were more concerned with gaining efficient translation outcomes than with engaging in the analytical processes behind translation decision-making. The translation process consists of three major phases: comprehension of the source text, generation of the target text, and an extra factor X (Schaeffer and Carl, 2013). According to Zhou and Hu (2019), the exertion of translation strategic competence is the essence of factor X. Despite effectively tackling the basic “comprehension” and “generation” tasks, AI may not thoroughly substitute the translator’s engagement in strategic decision-making during translation. Previous studies have shown that, when learners rely excessively on AI-generated outputs, technological convenience may reduce chances for deep cognitive processing (Stadler et al., 2024; Gerlich, 2025a; Gerlich, 2025b). Furthermore, AI may lead to superficial cognition and excessive reliance, thereby undermining students’ critical thinking and the ability to engage in deeper processing (Wang and Wang, 2024; Zhou and Hou, 2024). Furthermore, this explanation was supported by the qualitative findings. Some students worried about overly relying on AI tools. As demonstrated by classroom observations, lots of students did not evaluate extensively, but passively accepted AI-generated translations. Particularly, when AI offered apparently high-quality translations, students occasionally focused more on the final product, rather than the reasoning underlying certain translation selections. Under such practices, learners may have fewer opportunities to independently examine source texts, compare alternatives, and validate translation decisions, but all of them are crucial elements of cognitive engagement.

This finding suggests that AI-collaborative learning demands meticulously designed instructional settings to convert technological assistance into meaningful cognitive engagement. When AI outputs are considered just as answers or standards, learners’ opportunities to engage in independent reasoning may be reduced. On the contrary, if guidance is provided to learners to doubt, compare, and adjust AI-generated translations, AI can function as a catalyst for deeper learning.

From the perspective of instructional scaffolding, significant learning effects could not be generated by merely relying on technological scaffolding. In addition, peer cooperation and teacher intervention are required (Wang et al., 2026). Both individual and peer elements should cooperate to propel cognitive engagement. Although the intervention designed in this study incorporated comparison activities and group discussion, the qualitative data showed that the collaborative mechanism was not always thoroughly activated. According to the findings, less engaged students’ participation in discussion was less frequent. By contrast, highly engaged students often attached importance to individual comparison with AI outputs. Both kinds of students avoided true, in-depth cognitive reflection. That is to say, collaborative scaffolds alone are inadequate. Students need clear guidance and a well-defined framework of accountability in order to engage in meaningful interaction with their peers and move beyond individual, technology-driven learning behaviors.

In summary, AI integration has a complicated relationship with learner engagement. Although AI-collaborative translation instruction helps effectively motivate students’ behavioral engagement and positive emotional experiences, extra pedagogical scaffolding is required for deeper cognitive involvement. Therefore, future AI-collaborative translation courses should design tasks that require learners to attempt independent translation before AI consultation, critically compare human and AI-generated translations, and clearly justify revision decisions, so as to underscore their active agency. Meanwhile, such methods can help position AI as a solution provider and a learning partner for supporting reflective and tactic translation development.

## 6 Conclusion

This study has examined the role of AI-collaborative translation workshops in fostering translation competence and enhancing learner engagement. According to the results, the behavioral and emotional engagement of experimental group students was significantly higher, and their translation performance in textual faithfulness and linguistic appropriateness also improved. However, the intervention failed to significantly strengthen students’ capacity to effectively express cultural connotations or promote their cognitive engagement. As indicated by the findings, students’ higher-order translation competence and cognitive engagement could not be automatically cultivated through the only use of AI tools. Rather, the integration of AI into pedagogical processes to stimulate learner decision-making, reflective thinking, and critical assessment determines the educational value of AI.

Theoretically, this study transcends a technology-oriented perspective that regards AI as a tool for improving learning outcomes, and contributes to the new field of AI-collaborative pedagogy. Research findings indicate that the value of human-AI collaboration lies in its capacity to reshape learners’ engagement processes and support the development of translation competence through guided interaction with AI. In particular, this study suggests that AI should not substitute for learners’ translation decisions and professional judgment, but rather serve as a cognitive support mechanism to facilitate students’ abilities to compare, reflect, and engage in strategic problem-solving.

### 6.1 Pedagogical implications

The findings provide some pedagogical implications. First, simply providing students with opportunities to use AI tools is insufficient to achieve effective learning outcomes. To a certain extent, the AI-collaborative translation workshop helps improve students’ behavioral and emotional engagement. However, students’ improvement in cultural interpretation and cognitive engagement remains limited. In other words, students need clear guidance on how to critically evaluate AI-generated translations. Due to AI’s limitations in handling culturally rooted and context-dependent tasks (Wang, 2025; Sun, 2023), teachers should design activities to help students identify, elaborate, and revise AI-generated translations, so as to nurture their critical AI literacy. Such kinds of practices will enable AI to transform from an “answer provider” to into an object of “critical reflection” (Wang and Zhang, 2025; Qin and Ma, 2025).

Second, instructional design should give priority to students’ active cognitive engagement in the whole translation process. Research findings indicate that some students may rely too heavily on AI-generated outputs and fail to fully engage in the reasoning process underlying the translation choices. In response, a structured, segment-based translation workflow can be adopted by teachers: after independently completing a draft translation, students consult the translation suggestions from AI, compare different translations, illustrate their revision rationale, and finalize their work. Before referring to AI, students should first consider their own translation strategies and solutions and firmly control the final translation (Mu and Zheng, 2025). Encouraged by such practices, students will regard AI translations as analytical resources, so that deep cognitive engagement can be promoted.

Third, scaffolding support provided by teachers and peers is essential for effectively integrating AI into teaching. Even though collaborative learning activities were involved in this intervention design, qualitative findings show that peer interactions were not completely realized. In the future classroom practices, a more explicit framework for collaboration should be established (e.g., assigning discussion roles, requiring evidence-based explanations of translation choices, and incorporating reflective activities following AI-collaborative translation tasks). Sufficient instructional scaffolding can allow AI to complement learners’ cognitive efforts, so that they will be able to develop higher-level translation judgment and strategic competence.

### 6.2 Limitations

Several limitations of this study should be acknowledged. Firstly, this study employed a quasi-experimental design involving two intact classes, and participants were not randomly assigned to the experimental and control groups. Due to possible pre-existing differences between groups, the observed outcomes may be affected. Secondly, given that this intervention combined the use of AI tools with collaborative learning activities, the independent effects of each could not be fully separated. The combined influence of both instructional elements may have triggered the observed improvements. Thirdly, the sample size was relatively small, restricting the findings’ generalizability. Lastly, the intervention lasted for only a short period of time, so that the delayed effects of the AI-collaborative translation workshop on translation competence and learner engagement could not be examined.

### 6.3 Future research

To address these limitations, future research could employ more rigorous research designs and expand the scope of investigation. Firstly, different comparison groups (e.g., AI-only group, collaboration-only group, and AI-collaborative group) could be established in experimental studies to elaborate on the concrete contributions of different instructional elements. Secondly, the delayed effects of AI-collaborative instruction on the development of translation competence and learner engagement could be explored through longitudinal studies. Thirdly, future research could examine individual differences and the psychological mechanisms behind successful human-AI collaborative learning by incorporating learner relevant variables, such as growth mindset, academic self-efficacy, and AI literacy. Such investigations can further advance theoretical understanding and pedagogical practices in AI-collaborative translation education.

## Data Availability

The datasets presented in this article are not readily available because the dataset has been fully anonymized. It may only be used for academic research purposes. Commercial use and secondary distribution are prohibited. Requests to access the datasets should be directed to WX, 273033643@qq.com.
