# Supplementary material for: The effects of AI-collaborative translation workshop on translation competence and learner engagement: an empirical study in a Chinese university translation course
Source: Front Psychol. 2026 Aug 20;17:1918404. doi: 10.3389/fpsyg.2026.1918404 (PMC13537988; doi:10.3389/fpsyg.2026.1918404)
Supplement: Supplementary file 1 [file Supplementary_file_1.pdf]

Supplementary Material

1 Supplementary Tables

Table 1

Internal consistencies of agentic engagement scale.

Dimensions	Number of items	Cronbach's alpha at pretest	Cronbach's alpha at posttest
Behavioral Engagement	4	.850	.852
Agentic Engagement	5	.796	.760
Cognitive Engagement	4	.783	.916
Emotional Engagement	4	.725	.853

Table 2

Profile of interview participants.

Engagement performance group	Treatment group	Name	Gender
High	Experimental	Meng	Female
High	Experimental	Wei	Female
High	Control	Yu	Female
High	Control	Xue	Male
Low	Experimental	Zhang	Female

Low	Experimental	Xin	Female
Low	Control	Qing	Female
Low	Control	Ke	Male

Table 3

Pre- and post-test results of translation proficiency.

Dimensions	Group	N	Pre-test M	Pre-test SD	Post-test M	Post-test SD	ANCOVA Sig.	Effect size (partial η^2)
Overall performance	CG	34	6.96	2.80	8.31	2.94	.003	.124
	EG	35	6.26	2.20	8.96	3.05		
Textual faithfulness	CG	34	3.21	1.12	3.54	1.05	.003	.123
	EG	35	2.73	0.86	3.74	1.30		
Linguistic appropriateness	CG	34	2.47	0.98	3.00	0.86	.036	.065
	EG	35	2.10	0.77	3.03	0.84		
Effectiveness of cultural transmission	CG	34	1.31	0.94	1.77	1.19	.229	.022
	EG	35	1.41	0.82	2.11	1.16		

Note. CG=control group, EG=experimental group

Table 4

Pre- and post-test results of learner engagement.

Dimensions	Group	N	Pre-test	Post-test	ANCOVA Sig.	Effect size (partial η^2)

			M	SD	M	SD		
Overall engagement	CG	33	2.44	0.47	4.01	0.32	.013	.093
	EG	34	2.82	0.29	4.32	0.33		
Behavioral engagement	CG	33	2.52	0.71	3.80	0.63	.000	.244
	EG	34	2.81	0.40	4.46	0.42		
Cognitive engagement	CG	33	2.49	0.72	4.28	0.65	.868	.000
	EG	34	2.68	0.53	4.21	0.63		
Emotional engagement	CG	33	2.47	0.53	3.98	0.75	.013	.092
	EG	34	2.82	0.55	4.49	0.46		
Agentic engagement	CG	33	2.27	0.53	3.99	0.67	.650	.003
	EG	34	2.99	0.61	4.11	0.63		

Note. CG=control group, EG=experimental group

2 Supplementary Appendixes

Appendix A Classroom observation checklist

Observer: __________________

Date: __________________

Class/Group: □ Experimental □ Control

Lesson Topic: __________________

Number of students present: ______

Time	Behavioral engagement	Emotional engagement	Cognitive engagement	Field notes

Appendix B Cultural-text translation assessment rubrics

Scoring aspects	Textual faithfulness (6)	Linguistic appropriateness (4.5)	Effectiveness of cultural transmission (4.5)
Scores	Description		
Excellent (13.5–15)	5.5–6: Demonstrates a profound understanding of the source text. Accurately conveys all core information and implicit logic with no omissions or distortions. Rhetorical structure is perfectly preserved or adapted.	4–4.5: The target text is virtually indistinguishable from a native text. Vocabulary is precise and sophisticated; syntax is varied and natural. No grammatical errors.	4–4.5: Cultural elements are handled with exceptional sensitivity. Nuances are effectively conveyed, achieving a perfect balance between foreignization and domestication. The translation bridges cultural gaps seamlessly.
Good (12-13)	5–5.5: Addresses the writing task effectively. Conveys almost all key information accurately. Minor deviations in nuance do not affect the overall message. Logical flow is clear.	3.5–4: Displays a strong command of the target language. Expression is fluent and mostly idiomatic. Very few minor errors that do not impede communication.	3.5–4: Most cultural references are translated appropriately. The translator shows good awareness of cultural differences, though some subtle nuances might be slightly simplified.
Fair (9-11.5)	3.5–5: Adequately addresses the task but misses some details or misinterprets minor points. The general meaning is conveyed, but logical connections may be occasionally loose.	2.5–3.5: Language is generally correct but lacks variety. Some awkward phrasing or "translationese" is present. Errors occur but the text remains intelligible.	2.5–3.5: Basic cultural meanings are conveyed, but deeper cultural connotations are often lost or literalized. The translation feels somewhat culturally distant or overly explanatory.
Poor (6-8.5)	2–3.5: Significant omissions or mistranslations of key information. The logic of the source text is frequently distorted.	1.5–2.5: Frequent grammatical errors and poor word choices interfere with readability. Sentence structures are repetitive	1.5–2.5: Cultural barriers are not overcome. Misunderstanding of cultural context leads to inappropriate

	The translation fails to capture the main idea in several parts.	or broken. Strong influence of source language syntax (Chinglish).	translations. The target reader would likely be confused by cultural references.
Unqualified (0-5.5)	0–2: Fails to address the task. Major segments are missing or completely unrelated to the source text. Severe distortion of meaning makes the text incomprehensible.	0–1.5: Pervasive errors make the text unintelligible. Fundamental lack of control over grammar and vocabulary.	0–1.5: Complete failure to recognize or handle cultural elements. Offensive or nonsensical translations regarding cultural content.

Appendix C Coding Scheme

Theme	Categories		Examples
	First-level	Second-level	
Learner engagement	Behavioral engagement	Careful listening	Students listened attentively as the teacher introduced the course and AI tools; several raised questions about AI's role in translation.
		Sustained attention	Students focused on the explanation of serial-verb constructions; one asked how to make AI distinguish primary and secondary actions.
		Making effort	When the AI tool lagged, students refreshed repeatedly and manually adjusted the output instead of giving up.
		Working to full potential	A student tried using an infinitive to better convey the sequence of actions, going beyond the AI's parallel-verb version.
	Emotional engagement	Showing interest	Many students raised their hands to ask whether AI provides "correct" answers and if human revision is still necessary, showing strong curiosity.
		Enjoying the class	After completing the first AI-assisted exercise, most students said AI saved them a lot of time and appeared positive and relaxed.
		Enjoying new knowledge	When a student shared that adding "classroom language style" as a prompt improved AI output, others eagerly took notes.
		Positive emotional experience	During the final project sharing, students actively reflected on how AI helped them learn and emphasised the irreplaceable role of human proofreading.

Theme		Categories	Examples
		Connecting to prior knowledge	"The biggest gain this semester is learning to use AI for a draft, but we must understand the original text first; otherwise we won't even notice its mistakes."
		Integrating different ideas	Students compared multiple AI translations of complement structures, tried adverbial paraphrases, and explained the differences to their group members.
	Cognitive engagement	Making connections to tasks	"Here an infinitive works better than coordinate verbs."
		Creating own examples	After analyzing English modifier order, a student summarised the rule in her own notes and shared it with the group.

Appendix D A brief outline of the intervention Chinese-English course

Session	Phase	Content	AI-collaborative workshop
1	Introduction	Course description and introduction to the workshop	
2	Phase one: syntactic restructuring	Strategies for translating topic sentences into English	Compare multiple AI translations of topic-comment sentences and justify the best subject choice
3		Verb handling in serial verb and pivot constructions	Analyze and revise AI translations of serial-verb and pivot constructions, experimenting with different verb forms
4		Transformations of empty	Identify how AI handles empty objects and fronted patients;

		objects and fronted patients	rewrite by adding expletive “it” or changing voice
5	Phase two: structural supplementation	Strategies for translating attributives and modifiers	Compare human and AI ordering of multiple attributes; summarize English modifier sequence rules
6		Strategies for translating adverbials (structures)	Reorder AI-generated adverbials into natural English sequence
7		Strategies for translating complements and verb-complement structures	Generate multiple AI versions of complement structures and select the most appropriate English equivalent
8	Phase three: discourse translation	Strategies for dealing with cohesion devices	Revise AI translations of Chinese cohesive devices to avoid ambiguity
9		Strategies for transforming logical connection	Add explicit logical connectors to AI translations that omit them; discuss meaning changes with different connectives
10		Parataxis-hypotaxis shift	Convert a Chinese paratactic run-on sentence into a hypotactic English version using AI as a starting point
11		Text structure and coherence	Examine AI-translated paragraphs for given-new information flow and reorder sentences to improve coherence
12	Phase four: cultural adaptation	Techniques for translating Chinese idioms into English	Compare AI literal and free translations of idioms; decide when to add notes or use English equivalents
13		Techniques for translating culture-specific items	Propose improved translations for culture-specific items after evaluating AI output

14		Techniques for translating Chinese cultural thinking into English	Analyze AI translations of cultural allusions and paratactic thinking; balance cultural retention with readability
15		Final project	
16	Review	Review	
